# Twenty classic signs in neuroradiology: A pictorial essay

**DOI:** 10.4103/0971-3026.50835

**Published:** 2009-05

**Authors:** Govind B Chavhan, Manohar M Shroff

**Affiliations:** Department of Diagnostic Imaging, Hospital for Sick Children, University of Toronto, 555, University Avenue, Toronto, Ontario M5G 1×8, Canada

**Keywords:** Classic signs, education, neuroradiology

## Abstract

In this article we describe 20 classic signs in neuroradiology and provide illustrations of each; we also discuss the causes for their appearance, their reliability and sensitivity, and the differential diagnoses to be considered when they are encountered on imaging.

## Introduction

Classic signs in radiology, when invoked, immediately bring an image to mind and add confidence to the diagnosis of certain conditions. Familiarity with these signs helps in arriving at a diagnosis in day-to-day practice. In this pictorial essay we describe 20 classic signs in neuroradiology. We provide illustrations of these signs and discuss the pertinent features related to each sign. The focus of the discussion is on the cause of the appearance of these signs, the reliability and sensitivity of the signs, and the differential diagnoses to be considered when they are encountered on imaging.

## Ice-cream cone sign

**Figure 1 (A, B):**
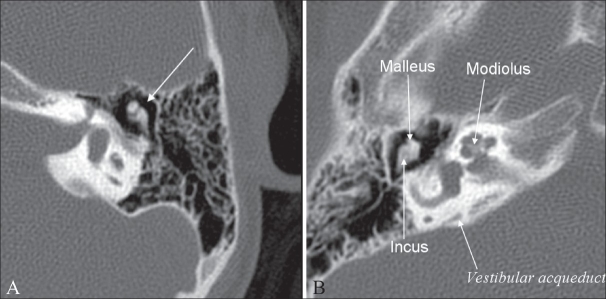
Ice-cream cone sign. Axial high-resolution CT scans of the temporal bone (A-left and B-right) show the ice-cream cone appearance of the incus and malleolus (long arrow in A). The different structures of the middle and inner ear are shown in B

The ice-cream cone sign represents the normal appearance of the malleus and incus on an axial high-resolution CT scan (HRCT) image of the temporal bone [Figure 1]. The ball (scoop) of the ice cream is formed by the head of malleus and the cone is formed by the body of the incus. The space between the ice-cream cone and the scutum is called Prussak's space.

## Empty delta sign

**Figure 2 (A–C):**
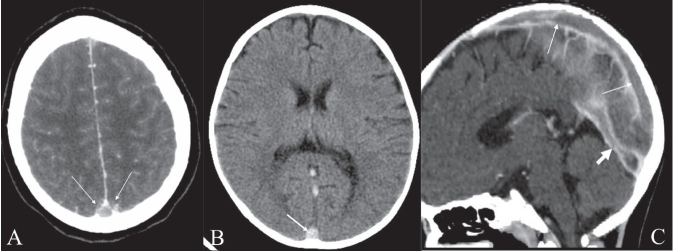
Empty delta sign. Postcontrast axial CT scan of the brain (A) shows the nonenhancing lumen of the superior sagittal sinus surrounded by enhancing dura (arrows). Nonenhanced axial CT scan of the brain (B) shows a hyperdense superior sagittal sinus (arrow) suggestive of thrombus within. This is called the ‘triangle sign.’ Sagittal reconstruction of a contrast-enhanced CT scan (C) shows filling defects in the superior sagittal sinus (long arrows) and in the straight sinus (thick arrow), suggestive of thrombus

This sign is created by a nonenhancing thrombus in the dural sinus surrounded by triangular enhancing dura as seen on cross-section. The sign, seen on contrast-enhanced CT scan images [Figure 2A and C], suggests dural sinovenous thrombosis. It is best seen on wider window settings. It is a reliable sign of sinus thrombosis but is seen only in 25–30% of these cases.[1] The use of multislice contrast-enhanced CT scan, with reconstructions into thinner slices in different planes, markedly improves the yield. The sign may not be seen in the early stage (less than 5 days) of thrombosis, as the fresh clot is hyperdense, or in the late stage (after more than 2 months), as numerous channels of recanalization develop in the thrombus after 2 months. The triangle sign [Figure 2B] and cord sign are signs of sinus thrombosis on nonenhanced CT scan. Both these signs represent clotted blood within the superior sagittal sinus, which is hyperdense on nonenhanced CT scan images. Hyperdense subarachnoid hemorrhage, subdural empyema, or hematoma surrounding the sinus may result in the ‘pseudodelta’ sign on unenhanced CT scan. In such cases, contrast enhancement will show enhancement in the delta. High tentorial insertion in children can simulate the empty delta sign.

## Dural tail sign

**Figure 3 (A–B):**
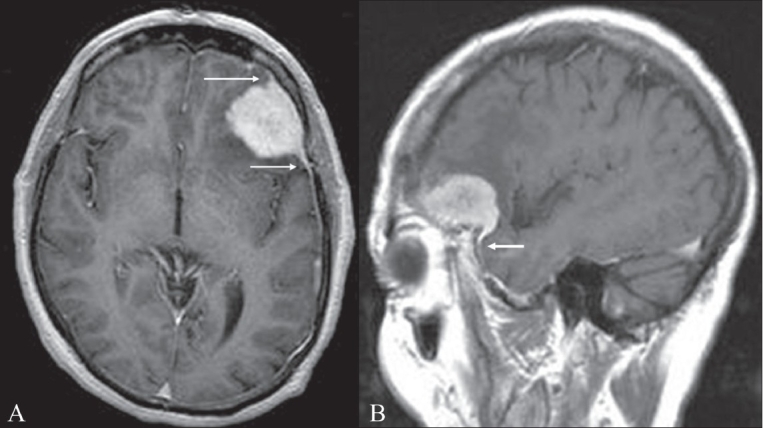
Dural tail sign. Axial (A) and sagittal (B) contrast-enhanced MRI images of the brain show an avidly enhancing lesion in the left frontal region. The lesion is dural based and there is thickening and enhancement of the dura, which tapers away from the mass (arrows). This thickening and enhancement of the dura adjacent to the mass is the ‘dural tail sign’ that is seen in meningiomas

This sign represents thickening and enhancement of the dura mater in continuity with a mass, which on MR images, gives the appearance of a tail arising from the mass [Figure 3]. The dural tail is thought to represent reactive change; however, it may also be due to tumor invasion.[2] Three criteria need to be met for a ‘positive’ dural tail sign: the tail should be seen on two successive images through the tumor, it should taper away from the tumor, and it must enhance more than the tumor. This sign has been traditionally considered as highly specific for meningioma. However, it is seen only in 60% of meningiomas and has also been reported in nonmeningiomatous lesions such as chloromas, primary CNS lymphomas, sarcoidosis, schwannomas, metastases, and syphilitic gummata.[2]

## Medusa head sign

**Figure 4 (A–C):**
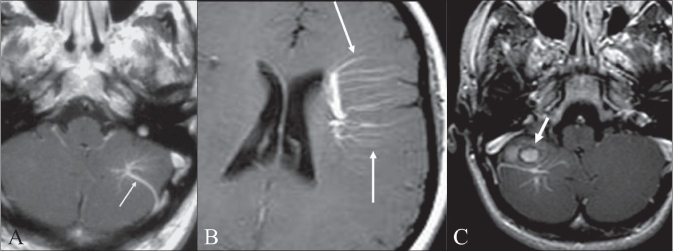
Medusa head sign. Postcontrast axial T1W MRI image of the brain (A) shows a evelopmental venous anomaly (arrow), with multiple, small, radiating veins forming a ‘Medusa head’ in the left cerebellar hemisphere. Postcontrast axial T1W MRI image of the supratentorial brain (B) shows a large developmental venous anomaly with multiple radiating veins (arrows) draining into it. Postcontrast axial T1W image of the brain (C) shows a large developmental venous anomaly and a round hyperintense lesion with a dark rim (arrow), suggestive of a cavernoma, anterior to it

The medusa head sign is seen in a developmental venous anomaly (DVA), where multiple tributaries arranged in a radial fashion drain into a larger vein [Figure 4A]. This sign is best seen on gadolinium-enhanced T1W images. DVAs are usually located in the juxtacortical and periventricular regions [Figure 4B] and are commonly seen in the frontal and parietal lobes and in the brachium pontis. DVA is considered a nonpathologic variation of venous drainage and, by itself, is usually not of any clinical significance.[3] However, it can occur in association with a cavernoma [Figure 4C]; it is seen in approximately 25–30% of cavernomas.[4]

## Lemon sign

**Figure 5 (A, B):**
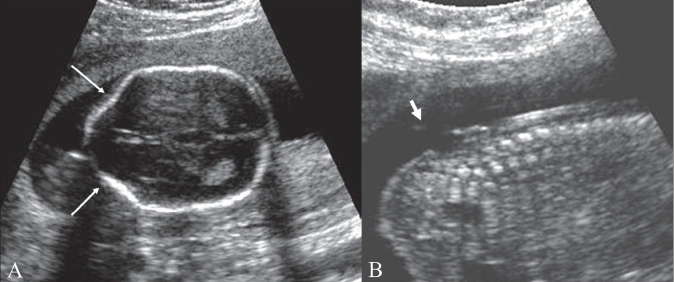
Lemon sign. Axial USG image of the fetal head at the level of the biparietal diameter (A) shows inward concavity of both frontal bones (arrows) instead of the normally seen convexity. This gives the head the appearance of a lemon. Sagittal USG image of the fetus (B) shows a meningomyelocele (thick arrow) in the lumbar region

This sign represents the loss of the normal convex contour of the frontal bones, with flattening or inward scalloping, seen on a transverse fetal sonogram obtained at the biparietal diameter level [Figure 5A]. It has a strong association with spina bifida and is very useful for detecting this condition [Figure 5B] before 24 weeks of gestation in high-risk patients. It has high sensitivity and specificity.[5] The lemon sign may disappear as gestational age advances and hence it is less reliable after 24 weeks. The sign is not specific for spina bifida and has also been seen in encephalocele, Dandy-Walker malformation with encephalocele, thanatophoric dysplasia, cystic hygroma, diaphragmatic hernia, corpus callosal agenesis, hydronephrosis, and umbilical vein varix.[6] Spina bifida is commonly associated with ventriculomegaly and Chiari II malformations. Other cranial markers include microcephaly; obliteration of the cisterna magna, with an absent cerebellum; and an abnormal anterior curvature of the cerebellum (‘banana sign’). A mild lemon sign may be normally seen and needs to be differentiated from a true sign. The lemon sign can also be falsely produced by angling the probe downward and anteriorly to include the orbit.

## Pancake brain

**Figure 6 (A, B):**
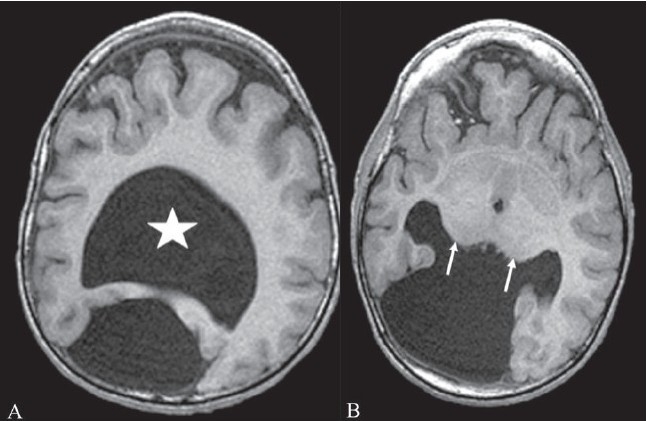
Pancake brain. Axial T1W MRI images (A, B) of the brain in alobar holoprosencephaly show fusion of the cerebral hemispheres, with a single ventricular cavity (star) in the center; this gives the brain the appearance of a pancake. Note the fusion of the thalami (arrows)

This sign describes the appearance of the abnormal brain in alobar holoprosencephaly (HPE). The appearance is caused by fusion of the cerebral hemispheres and the presence of a single ventricle in the center [Figure 6A and B].[7] HPE is a congenital malformation of the brain, with variable fusion of the cerebral hemispheres, diencephalon, basal ganglia, and thalami. In alobar HPE there is complete fusion and a monoventricle; it represents the most severe form of the malformation. The other two types are semilobar and lobar HPE. Lobar HPE is the least severe form. HPE is often associated with midline facial abnormalities, and the saying ‘the face predicts the brain’ holds true for HPE.

## Reversal sign

**Figure 7:**
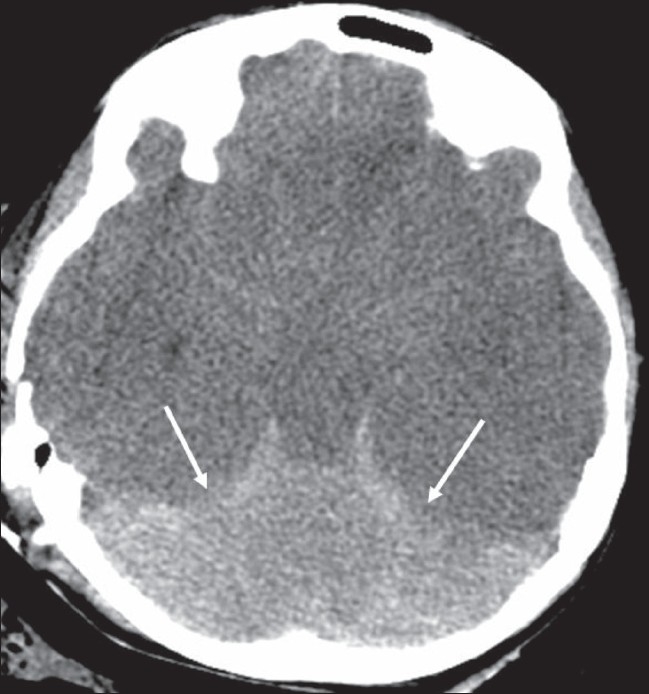
Reversal sign. Axial noncontrast CT scan of the brain in a child with severe head injury shows low attenuation of the cerebral hemispheres with complete loss of gray matter-white matter differentiation, small temporal horns, and effacement of the basal cisterns. The findings are suggestive of cerebral edema. There is relative preservation of the cerebellum (arrows) with hyperattenuation (‘white cerebellum’)

This sign is seen on CT scan images and represents a diffuse decrease in the density of the cerebral hemispheres, with loss of gray-white differentiation and a relative increase in the density of the thalami, brainstem, and cerebellum [Figure 7].[8] It is also known as the ‘white cerebellum sign.’ It is seen in severe head injury, birth asphyxia, drowning, status epilepticus, bacterial meningitis, and encephalitis. It represents anoxic/ischemic cerebral injury. It indicates irreversible brain damage and carries a poor prognosis.[8] One of the proposed mechanisms for the appearance seen in the reversal sign is distension of the deep medullary veins secondary to partial obstruction of venous outflow due to raised intracranial tension. Other theories that have been proposed include preferential flow to the posterior circulation and transtentorial herniation partially relieving the raised intracranial tension, leading to improvement in the perfusion of central structures such as the brainstem.[8]

## Hyperdense MCA sign

**Figure 8 (A, B):**
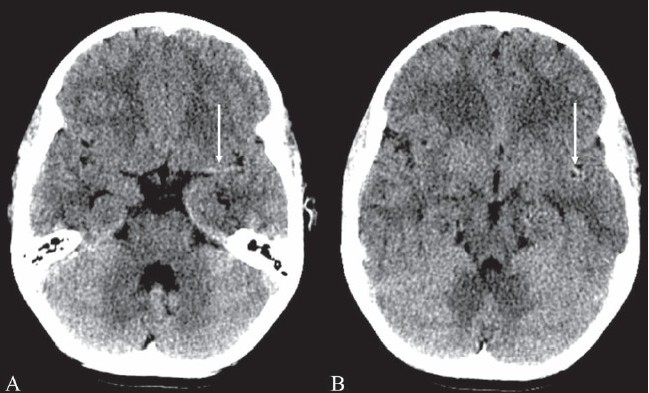
Hyperdense MCA sign. Noncontrast axial CT scans (A, B) of the brain in a patient with sudden-onset right hemiparesis show hyperattenuation of the left middle cerebral artery (arrows). This is suggestive of occlusion of the artery. This is one of the signs of acute stroke but does not necessarily represent infarction

This sign refers to the hyperdense middle cerebral artery (MCA) seen on nonenhanced CT scan images in acute stroke [Figure 8A and B]. Hyperdensity of the MCA is a result of acute clot formation within the artery. The sign is said to be seen usually within approximately 90 min of the event. It has a high specificity of almost 100% but a very low sensitivity of 30% in the diagnosis of acute stroke.[9] It is suggestive of occlusion of the MCA but does not necessarily represent infarction of the brain parenchyma. Two other signs seen on CT scan images in early acute stroke: ‘obscuration of lentiform nucleus’ and ‘insular ribbon sign,’ represent early infarction. It is important to recognize these early signs of acute stroke because ‘time is brain’—the time window for intravenous thrombolysis with tPA (tissue plasminogen activator) is 3 h, while it is 6 h for intra-arterial thrombolysis.

## Salt and pepper sign

**Figure 9 (A–C):**
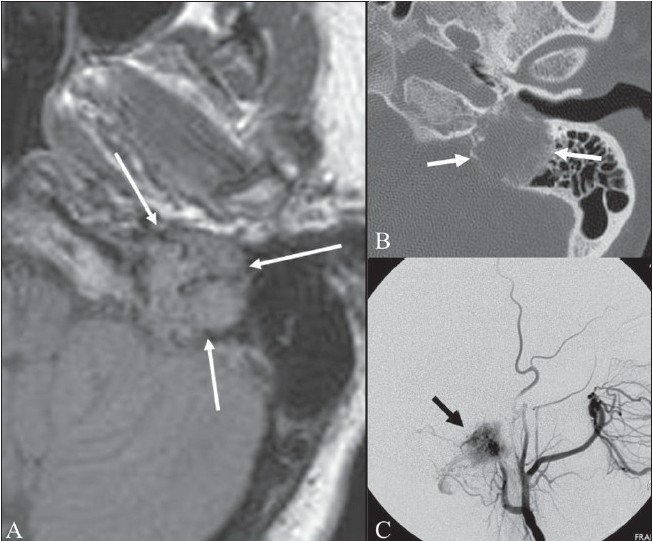
Salt and pepper sign. FLAIR axial MRI image of the left temporal bone shows an isointense mass (arrows) in the region of the jugular foramen. The mass is heterogeneous, with dark and slightly bright areas within it. Axial CT scan (B) in the same patient shows the mass causing destruction of the surrounding bone (arrows). An external carotid angiogram (C) shows tumor blush (black arrow) due to hypervascularity. These findings are suggestive of a glomus jugulare tumor of the jugular foramen

This sign is seen on MRI images in paragangliomas such as glomus tumors [Figure 9 A–C]. The sign is indicative of the hypervascularity of the mass. The ‘pepper’ represents multiple areas of signal void of vessels and the ‘salt’ represents the hyperintense foci due to slow-flow vessels or hemorrhages in these hypervascular tumors.[10] The sign is seen in tumors that are more than 1 cm in diameter. The sign is not specific for paragangliomas and has been reported in other hypervascular lesions such as metastatic hypernephroma and metastatic thyroid carcinoma. Four common locations of paragangliomas in the head and neck are the carotid body, the jugular foramen, along the path of the vagus nerve, and the middle ear.[10] Paragangliomas can be multiple and bilateral, especially in familial cases, and hence evaluation of the entire neck and of both sides is needed.

## Optic nerve tram-track sign

**Figure 10 (A, B):**
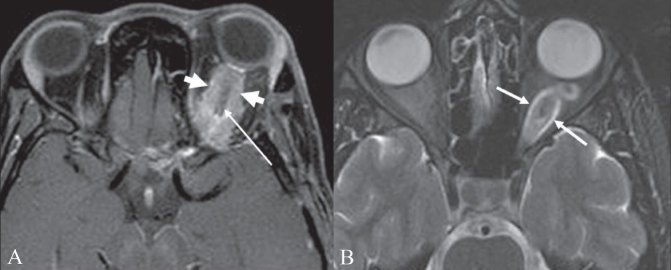
Optic nerve tram-track sign. Postcontrast T1W axial MRI image of the orbits (A) shows an enhancing mass (thick small arrows) around the left optic nerve. The optic nerve (thin long arrow) itself is not enlarged and is nonenhancing. This is suggestive of a mass, such as a meningioma, arising from the optic nerve sheath. Axial T2W MRI image of the orbits in a different patient (B) shows fusiform enlargement of the left optic nerve itself (arrows), in a case of optic nerve glioma

A tram-track sign is composed of two enhancing areas of tumor separated from each other by the negative defect of the optic nerve.[11] It is seen on contrast-enhanced CT scan and MRI images [Figure 10A], in optic nerve sheath meningioma. The sign helps distinguish between optic nerve sheath meningioma and optic glioma. Optic glioma arises from glial cells within the optic nerve and there is no clear separation between the nerve and the tumor; hence the tram-track sign is not seen in optic gliomas [Figure 10B]. Calcification may be seen in optic nerve sheath meningiomas in 20–50% of cases and hence the tram-track sign may be seen on nonenhanced CT scan images as a linear calcification around the nerve, but this is less common. Optic nerve meningioma is usually seen in women in the third to fifth decades of life and in children with neurofibromatosis type 2, where it may be bilateral. The tram-track sign is not specific for meningiomas and has also been described in orbital pseudotumor, perioptic neuritis, sarcoidosis, leukemia, lymphomas, metastases, perioptic hemorrhages, and Erdheim-Chester disease (a rare non-Langerhans cell histiocytosis).[11]

## Bare orbit sign

**Figure 11 (A–C):**
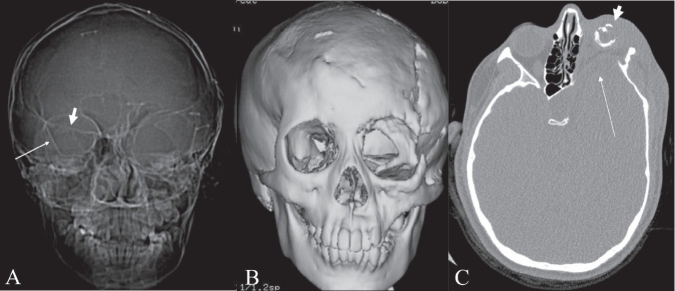
Bare orbit sign. Frontal CT scan scannogram of the head (A) shows a bare and large orbit on the left, with absence of the greater wing of the left sphenoid bone, in this patient with neurofibromatosis type 1. Note the normal lesser (thick small arrow) and greater (thin long arrow) wings of the sphenoid on the right side. Frontal surface-shaded display, 3D view of the skull (B) shows a large left orbit with dysplasia of the sphenoid wing. Axial CT scan image (C) shows anterior herniation of the left temporal lobe (thin long arrow) because of the dysplastic sphenoid bone. The left globe is small and calcified (phthisis bulbi) (thick short arrow) due to plexiform neurofibromatosis of the left orbit

The bare orbit sign represents the appearance of the orbit on a frontal radiograph of the skull and is so called because of the absence of the innominate line, which is the projection of the greater wing of the sphenoid bone [Figure 11A and B].[12] This sign is seen in sphenoid wing dysplasia in neurofibromatosis type 1 (NF1). In addition to the absence of the innominate line, there is egg-shaped enlargement of the anterior orbital rim, a bony defect in the posterior orbit, and anteroposterior enlargement of the middle cranial fossa.[13] Sphenoid dysplasia is a distinctive bone lesion seen in 5–10% of cases of NF1[12] and is used as one of the National Institute of Health (NIH) diagnostic criteria for NF1. Sphenoid wing dysplasia associated with pulsatile exophthalmos, expansion of the temporal fossa, and herniation of the temporal lobe into the orbit have been described as the ‘cranio-orbital-temporal’ subtype of NF1 [Figure 11C].[12] The exact etiology of the sphenoid dysplasia is not clear. It could be part of the syndrome, with primary dysplasia as one of the components, or the changes in the sphenoid bone could be secondary to the presence of an orbital plexiform neurofibroma or other masses that are commonly seen in association with sphenoid dysplasia.[13]

## Moya moya appearance

**Figure 12 (A, B):**
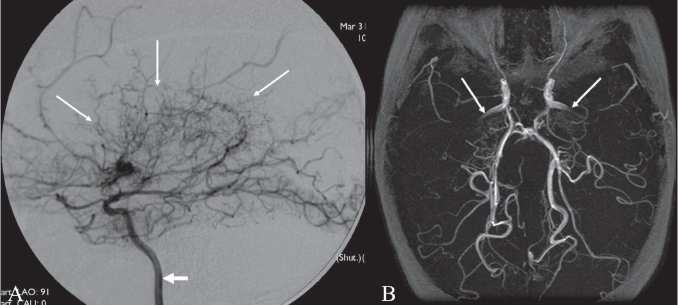
Moya moya appearance. Lateral anterior oblique view (A) of an internal carotid artery (thick short arrow) angiogram shows multiple, small, tortuous collateral vessels in the distribution of the middle cerebral artery (arrows), suggestive of the moya moya (puff of smoke) appearance. Axial view of the MRI angiogram (B) shows complete occlusion of the middle cerebral arteries bilaterally. Arrows indicate the internal carotid arteries

Moya moya is a Japanese term that means ‘puff of smoke.’ It represents the angiographic appearance of basal telangiectasias, which consist of dilated collateral branches of the lenticulostriate and thalamostriate arteries [Figure 12A and B].[14] It is usually seen in the anterior circulation in association with internal carotid artery stenosis. When the moya moya appearance is seen along with idiopathic occlusion of the internal carotid arteries it is called moya moya disease; when the occlusion is secondary to some other disease it is called moya moya syndrome. Causes of moya moya syndrome include NF1, sickle cell disease, bacterial meningitis, polyarteritis nodosa, radiation therapy, tuberculosis, and atherosclerosis. Histopathology of occluded arteries in moya moya disease shows endothelial hyperplasia and fibrosis without inflammatory reaction.[14] Children with moya moya usually have ischemia or infarction, while adults with moya moya usually have hemorrhage. The treatment of moya moya includes anticoagulation, hypertransfusion, encephalo-duro-arterio-synangiosis (EDAS), anastomosis of the superficial temporal artery with the intracranial arteries, and sympathectomy or cervical ganglionectomy.

## Hot nose sign

**Figure 13:**
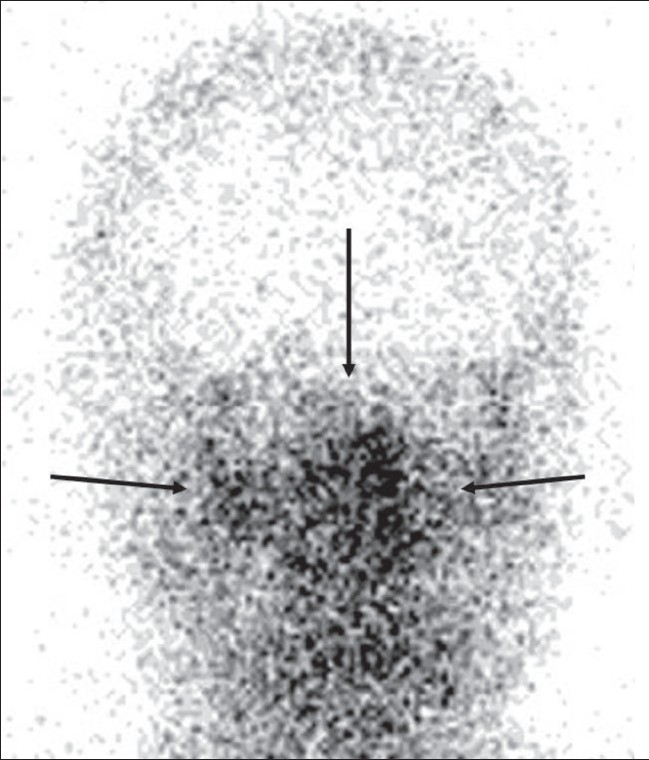
Hot nose sign. Frontal view of a radionuclide scan shows increased tracer activity in the nasopharyngeal region (arrows) suggestive of increased blood flow. Obstruction of the internal carotid artery from any cause leads to increased flow in the external carotid artery and increased tracer activity in the nasopharyngeal region (hot nose)

Hot nose sign represents increased perfusion in the nasopharyngeal region on radionuclide scans [Figure 13].[15] The hot nose sign is commonly associated with brain death and serves as a secondary sign of brain death when intracerebral perfusion is absent. Normally, radionuclide activity in the nasopharyngeal region is either not appreciated or seen only in the venous phase. Increased and early radiotracer activity in this region is seen in occlusion of one or both internal carotid arteries (ICA) from any cause, with consequent increased flow in the external carotid artery. Apart from mechanical occlusion, functional occlusion of the ICA due to elevated intracranial pressure in conditions such as cerebrovascular accident, transient ischemic attack, subdural hematoma, and hepatic encephalopathy can also produce the hot nose sign. The sign can be seen in 52% of patients with brain death.[15] The clinical utility of the sign has been questioned as it lacks sensitivity and specificity; moreover, a radionuclide study to confirm brain death is only performed rarely.

## Eye-of-the-tiger sign

**Figure 14:**
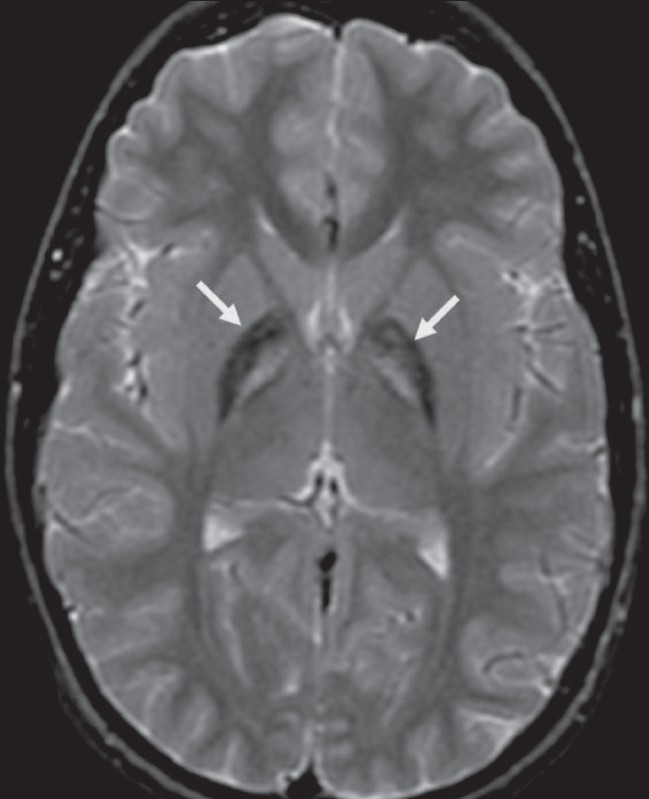
The eye-of-the-tiger sign. Axial T2W MRI image of the brain shows hypointensity of the globus palladi (arrows). There is relative hyperintensity of the central part, giving the globus palladi the appearance of the eyes of a tiger. This appearance is seen in Hallervorden-Spatz syndrome

This sign represents marked low signal intensity of the globus palladi on T2W MRI images. This low signal surrounds a central, small hyperintense area, producing the eye-of-the-tiger appearance [Figure 14].[16] The sign is seen in what was once known as Hallervorden-Spatz (HS) syndrome but is now called neurodegeneration with brain iron accumulation (NBIA) or pantothenate kinase II (PANC2)-associated neurodegeneration.[17] The marked low signal intensity of the globus palladi is a result of excessive iron accumulation and the central high signal is attributed to gliosis, increased water content, and neuronal loss with disintegration, vacuolization, and cavitation of the neuropil.[16] Iron levels in blood and CSF are normal. The HS syndrome is a neurodegenerative disorder associated with extrapyramidal dysfunction and dementia. It is a neuroaxonal dystrophy, with the pathologic triad of iron deposition, axonal spheroids, and gliosis in the globus pallidi. MRI is important for differentiating HS syndrome from infantile axonal dystrophy, which does not show iron deposition. Mutation of the gene for pantothenate kinase 2 is the cause for the syndrome.[17] The sign can be seen in other extrapyramidal Parkinsonian disorders such as cortical-basal ganglionic degeneration, Steele-Richardson-Olszewski syndrome, and early-onset levodopa-responsive Parkinsonism.[16]

## Molar tooth sign

**Figure 15 (A, B):**
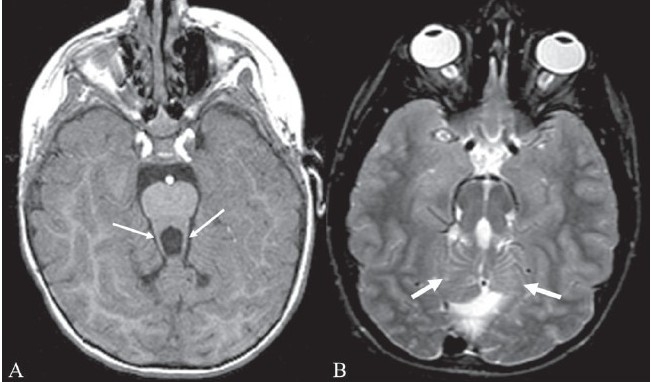
Molar tooth sign. Axial T1W MRI image of the brain (A) in a child with Joubert syndrome shows elongated superior cerebellar peduncles (arrows) giving the midbrain and superior peduncles the appearance of a molar tooth. Axial T2W image of the brain (B) in another child shows the molar tooth appearance of the midbrain and superior cerebellar peduncles. There is also hypoplasia of the cerebellar vermis (arrows)

This sign represents the abnormal appearance of the superior cerebellar peduncles at the level of the midbrain on axial CT scan or MRI images [Figure 15A and B]. The sign is produced by abnormal anteroposterior orientation of the cerebellar peduncles resembling the roots of a molar tooth. It is seen in Joubert syndrome (cerebellar vermian dysgenesis) and is associated with lack of normal crossing of the superior cerebellar peduncular fiber tracts in the midline.[18] Joubert syndrome is an autosomal recessive disorder characterized by cerebellar vermian hypoplasia with a midline cleft. Other findings that can be seen in the syndrome include microcephaly, dysmorphic facies, retinal dystrophy, tongue protrusion, congenital heart disease, corpus callosal agenesis, and unsegmented midbrain tectum. Imaging features in the brain include absence of vermis; thickening and reorientation of the superior cerebellar peduncles; and deformities of the fourth ventricle, such as a ‘bat wing’ appearance. The molar tooth sign is considered pathognomonic of the syndrome and is seen in 85% of cases.[18] Clinically, children with Joubert syndrome have mental retardation, hypotonia, abnormal eye movements, and abnormal breathing.

## Stripe sign/ tigroid pattern

**Figure 16 (A, B):**
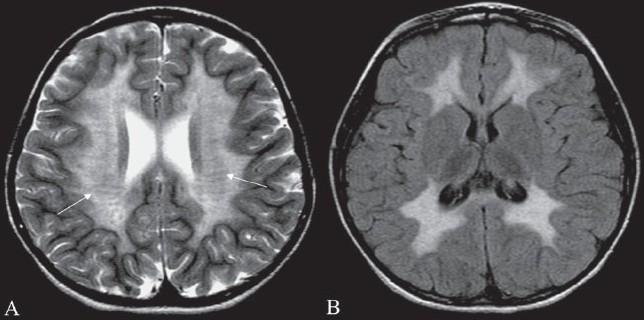
Tigroid pattern. Axial T2W image of the brain in a child with metachromatic leukodystrophy shows symmetric, increased signal intensity of the white matter, with sparing of the subcortical U fibers. Linear low signal intensity areas radiating away from the ventricular margin (arrows) represent areas of white matter around the vessels that have been spared from the process of demyelination. These low signal linear areas within the hyperintense white matter resemble the skin of a leopard and hence the term ‘tigroid’ pattern. FLAIR axial MRI image (B) shows symmetric hyperintensity of the white matter sparing the subcortical U fibers

This is seen as linear hypointensities radiating from the ventricular margins within hyperintense periventricular white matter and the centrum semiovale on T2W MRI images [Figure 16A]. The sign represents a specific pattern of demyelination, with sparing of perivascular white matter.[19] The spared perivascular white matter is seen as dark spots or dark linear areas against a background of bright affected white matter, giving the appearance of the skin of a leopard. The sign is also called the ‘leopard skin sign.’ It is seen in metachromatic leukodystrophy (MLD). MLD is an autosomal recessive disorder with deficiency of the lysosomal enzyme arylsulfatase, leading to accumulation of sulfatides in the brain, peripheral nerves, kidneys, liver, and gall bladder. Arysulfatase is required for metabolism of sulfatides, one of the essential constituents of the myelin sheath.[19] MLD can be of three types: late infantile, juvenile, and adult. Late infantile is more common and usually presents between 12 and 18 months of age. Clinically, children present with peripheral neuropathy and changes in intellect, speech, and coordination. The disease is progressive, with gait abnormality, quadriplegia, decerebration, and death by the age of 6 months to 4 years. The diagnosis is confirmed by finding low levels of arylsulfatase in peripheral white blood cells and urine. MRI shows symmetric increased signal in the periventricular white matter, with initial sparing of the subcortical U fibers [Figure 16B]. No enhancement is seen in the brain tissue unlike in adrenoleukodystrophy. However, cranial nerve enhancement has been reported recently in MLD.[20]

## Tau sign

**Figure 17 (A, B):**
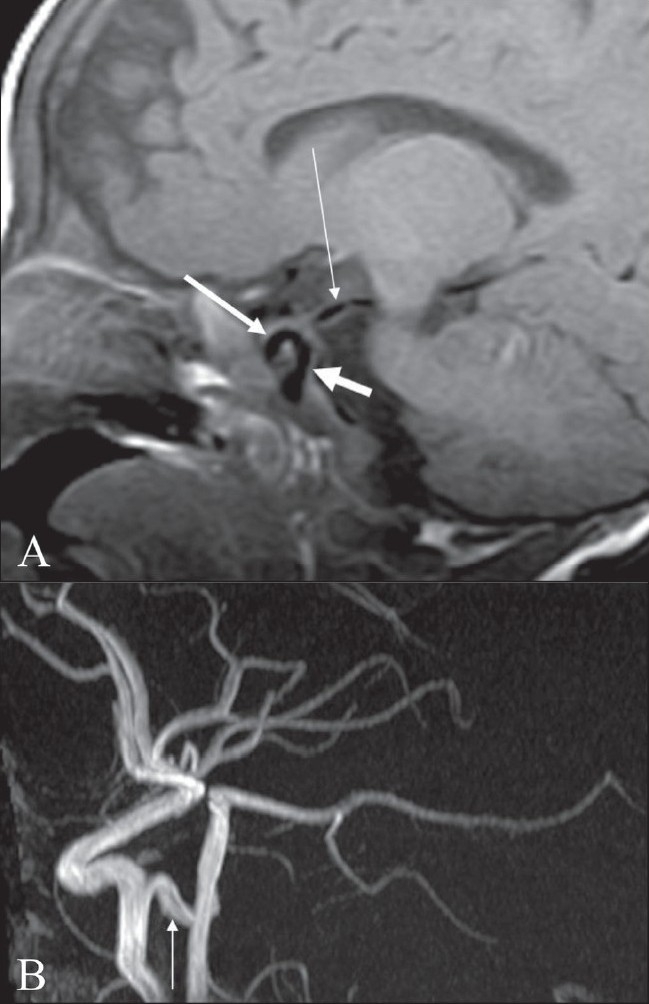
Tau sign. Sagittal T1-W image of the brain shows (A) flow voids of the internal carotid artery (ICA) in the precavernous segment (thick short arrow), in the cavernous segment (medium-sized arrow), and a persistent trigeminal artery (thin long arrow). Together, these flow voids form the Greek letter ‘τ’ (tau). Sagittal view of the MRI angiogram shows the persistent trigeminal artery (arrow) arising from the ICA and joining the basilar artery in its mid segment

The tau sign represents the appearance of the presellar internal carotid artery (ICA) when a persistent trigeminal artery (PTA) originates from it, on a T1W sagittal MRI image [Figure 17A]. The configuration of the flow void in the presellar segment of the ICA with the PTA arising from it, resembles the Greek letter ‘τ’ (tau). The sign is suggestive of a PTA.[21] The PTA arises from the ICA as it exits the carotid canal and enters the cavernous sinus [Figure 17B]. It joins the distal third of the basilar artery between the origins of the anterior, inferior, and superior cerebellar arteries. A PTA can be of two types: 1) the artery may supply the entire vertebrobasilar system distal to the anastomosis or 2) the anastomosis may mainly supply the superior cerebellar arteries bilaterally. PTA can be associated with aneurysms, arteriovenous malformations, moya moya disease, and other persistent carotid-vertebrobasilar anastomosis.[21] Other persistent arteries that are responsible for communications between the carotid and vertebrobasilar systems are persistent hypoglossal and otic arteries.

## Radial bands sign

**Figure 18 (A, B):**
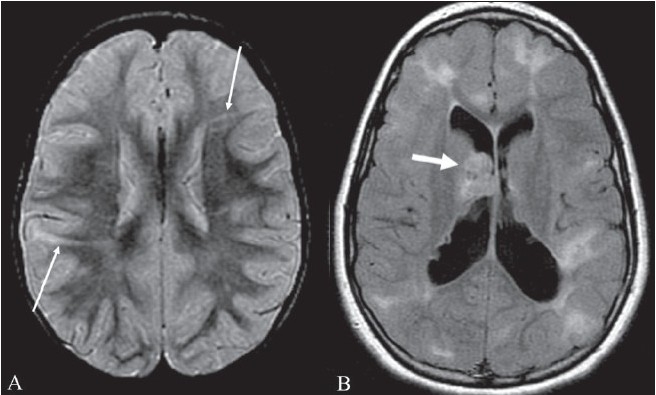
Radial band sign. Proton density-weighted axial image of the brain (A) in a patient with tuberous sclerosis shows hyperintense bands in the white matter radiating from the ventricular margin to the subcortical region (arrows). FLAIR axial MRI image (B) shows irregular hyperintense areas in the subcortical white matter suggestive of subcortical tubers. A large hyperintense nodule is seen in the region of the foramen of Monroe, representing a subependymal giant cell astrocytoma (thick arrow)

Radial bands are linear or curvilinear areas of abnormal signal intensity radiating from the periventricular region to the subcortical region on MRI images [Figure 18A].[22] These radial bands are seen in tuberous sclerosis and are best appreciated on a FLAIR sequence and on images acquired with magnetization transfer. It is thought that the radial bands represent abnormal migration of dysplastic stem cells along the course of the radial glial neuronal unit. Radial bands are hypointense to isointense on T1W images and hyperintense on T2W images in adults. They are hyperintense to unmyelinated white matter on T1W images and isointense to hypointense compared with white matter on T2W images in neonates and young children. The abnormal signal intensity is presumably due to lack of normal myelination and differences in cellular or interstitial fluid content compared to normal brain parenchyma. Visualization of radial bands is thought to be specific to tuberous sclerosis. Tuberous sclerosis is a common neurocutaneous syndrome characterized by the clinical triad of epilepsy, mental retardation, and adenoma sebaceum. Other intracranial manifestations include subependymal nodules, subependymal giant cell astrocytoma, cortical tubers, and white matter abnormalities [Figure 18B].

## Harlequin appearance

**Figure 19 (A–C):**
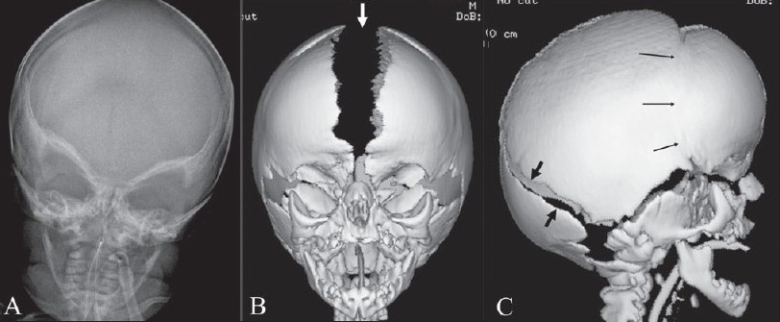
Harlequin appearance. Frontal view of the skull (A) in a child with Apert syndrome shows elevated superolateral angles of both orbits giving the appearance of a ‘harlequin mask.’ Frontal (B) and lateral (C) surface-shaded display 3D CT views of the skull show the harlequin appearance of the orbits. The sagittal suture (arrow in B) and the lambdoid suture (black arrows in C) are wide open. The coronal suture (thin arrows in C) is fused, which is suggestive of coronal craniosynostosis

Harlequin appearance of the orbit represents the elevation of the superolateral angle of the orbit along with a flat frontal bone on a plain radiograph [Figure 19A–C].[23] It is seen in coronal craniosynostosis, where the anteroposterior growth of the skull is limited. There is also relative increase in the transverse diameter of the skull, which is called brachycephaly. The orbit is shallow, the lesser wing of the sphenoid is elevated, and the greater wing is expanded. The innominate line (superior border of the greater wing of the sphenoid) appears as a dense ridge. The sign can be unilateral or bilateral.

## Hot cross bun sign

**Figure 20 (A, B):**
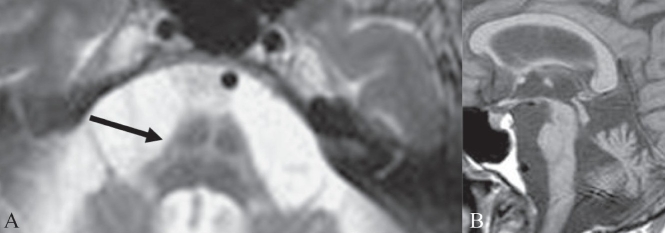
Hot cross bun sign. Axial T2W MRI image of the pons (A) in a patient with multiple system atrophy shows a hyperintense linear area forming a ‘cross’ (arrow) in the pons. Sagittal T1W image (B) shows atrophy of the cerebellum and cerebrum (prominence of sulci)

This sign refers to a cruciform hyperintensity of the pons on T2W MRI images [Figure 20A], which resembles a hot cross bun. It is typically seen in multisystem atrophy. The appearance is caused by the degeneration of pontine neurons and myelinated transverse pontocerebellar fibers in a cruciform pattern.[24] Multisystem atrophy is a neurodegenerative disorder in which there are features of Parkinsonism and/or cerebellar ataxia along with autonomic dysfunction. Multisystem atrophy may have predominant Parkinsonian features (MSA-P) or predominant cerebellar features (MSA-C). Other findings seen in multisystem atrophy are brainstem atrophy, putaminal atrophy, and abnormal signal in the middle cerebellar peduncles.[24] Other findings that can also be seen in Parkinsonism include hypointensity of the putamina, slit-like hyperintensity of the lateral putaminal border, and atrophy of the cerebellar vermis or hemispheres [Figure 20B]. Hot cross bun has also been reported in pure Parkinsonism.
